# Factors associated with changes in uptake of HIV testing among young women (aged 15–24) in Tanzania from 2003 to 2012

**DOI:** 10.1186/s40249-016-0180-3

**Published:** 2016-09-06

**Authors:** Michael J. Mahande, Rune N. Phimemon, Habib O. Ramadhani

**Affiliations:** 1 Kilimanjaro Christian Medical Centre, Moshi, Tanzania; 2 Department of Epidemiology and Biostatistics, Institute of Public Health, Kilimanjaro Christian Medical University College, Moshi, Tanzania

**Keywords:** HIV testing, Uptake, Young women, Tanzania

## Abstract

**Background:**

This study explored the factors associated with changes in HIV testing uptake among young women in Tanzania, based on an analysis of data from the 2003–2004 Tanzania HIV/AIDS Indicator Survey, and the 2007–2008 and 2011–2012 Tanzania HIV/AIDS and Malaria Indicator Surveys.

**Methods:**

The study population consisted of young women aged 15–24 years at the time of the survey. Multivariate decomposition analysis was used to assess factors associated with changes in HIV testing uptake between the 2003–2004 and 2007–2008 surveys, and between the 2007–2008 and 2011–2012 surveys.

**Results:**

HIV testing uptake among the study population was 7 % in 2003–2004, 31 % in 2007–2008 and 40 % in 2011–2012. The time period of the survey had a substantial effect on the uptake of HIV testing independent of other covariates. The characteristics that were significantly associated with a higher chance of HIV testing uptake across the surveys were age (20–24), education level (primary and secondary), ever being married, having at least one lifetime sexual partner, having a sexually transmitted infection or associated symptoms, and receiving antenatal care.

**Conclusions:**

Changes in the study participants’ characteristics in the 2003–2004 survey compared with the 2007–2008 survey were associated with a decrease in HIV testing uptake. Comparing the 2007–2008 survey with the 2011–2012 survey shows that the changes in the participants’ characteristics contributed to 22 % of the changes in HIV testing uptake, while 78 % of the changes were attributed to coefficients.

**Electronic supplementary material:**

The online version of this article (doi:10.1186/s40249-016-0180-3) contains supplementary material, which is available to authorized users.

## Multilingual abstracts

Please see Additional file 1 for translation of the abstract into the six official working languages of the United Nations.

## Background

The human immunodeficiency virus (HIV) remains a major global public health problem. Approximately 36.9 million people were estimated to be living with HIV/acquired immune deficiency syndrome (AIDS) in 2014 [1]. The majority (25.8 million, 70 %) are in Sub-Saharan Africa (SSA) [2]. Approximately 3.9 million young people aged 15–24 years in SSA are estimated to be living with HIV/AIDS, and of these, three quarters are young women [1]. In recent years, increased coverage of antiretroviral therapy (ART) has led to a decline in morbidity and mortality related to HIV and its associated opportunistic infections [3–5]. Studies have also shown a global decline in HIV incidence among the general population [6].

Tanzania, like other countries in SSA, continues to be challenged by the HIV epidemic. However, the prevalence of HIV has been reported to decline over time. According to the 2007–2008 Tanzania HIV/AIDS and Malaria Indicator Survey (THMIS), the prevalence of HIV declined from 7 % in 2004 to 6 % in 2008 [7]. More recently, the 2011–2012 THMIS indicated a further decline in the overall national HIV prevalence to 5.1 % in 2012 [8]. The proportion of men and women aged 15–45 years who have ever been tested for HIV and received results has increased from 27 and 37 % in 2008 to 47 and 62 % in 2012, respectively. However, this also indicates that a large proportion of people are unaware of their HIV status [9]. According to the 2011–2012 THMIS, the prevalence of HIV among young people aged 15–24 years was 11.2 % [10] which is higher than the national average. However, HIV prevalence was disproportionately higher among females as compared to males (6 % versus 4 %, respectively) [9]. Despite the high prevalence of HIV in this group, the survey showed that about 46.3 % of females were not aware of their HIV status [10].

HIV testing and counselling (HTC) is an integral component of HIV-preventive strategies. It is a gateway to care, treatment and support for people in need. Knowing one’s HIV status is critical in the fight against HIV [11, 12]. Infected persons may be counselled about how to live a healthy life with the disease, as well as increase their access to care and treatment (i.e. receive ART) [13]. Among the benefits of linking patients with HTC are prevention of mother-to-child transmission (PMTCT), preventing uninfected partners from becoming infected, improving quality of life, reducing morbidity and mortality related to opportunistic infections and reducing the frequency of hospitalisations [14, 15].

Previous studies using mathematical models have revealed that approximately 50 % of new HIV infections are from HIV-infected persons who are unaware of their HIV status [16]. HIV testing provides an opportunity for people to find out their HIV status. Therefore, knowing one’s HIV status may influence changes in personal behaviour, which makes individuals more vulnerable to becoming infected or infecting others with HIV, thus helping to reduce the spread of HIV [17, 18]. Delayed HIV testing makes it more difficult to prevent the spread of the infection [19]. While early diagnosis and treatment are associated with good disease outcomes [20], delayed diagnosis and treatment increase the disease burden and represent missed opportunities for prevention [21, 22].

Previous studies have reported on factors associated with testing for HIV among adolescent females in SSA. These include older age, HIV knowledge, having ever talked about HIV with parents or guardians, and having ever been pregnant [23]. Furthermore, perceived risk of contracting HIV, better knowledge about HCT, believing that someone in one’s school is infected, positive attitude towards HCT, availability of HCT centres, having a high level of HIV-related activities in schools and being sexually experienced, availability of HIV information at schools, and provision of counselling certificates recognised at higher education institutions have also been associated with uptake of HIV testing [24].

This study aimed to explore the trends in HIV testing uptake among young women aged 15–24 years between 2003–2004 and 2011–2012. We evaluated individual and contextual factors associated with changes in HIV testing uptake between the 2003–2004 Tanzania HIV/AIDS Indicator Survey (THIS), and the 2007–2008 and 2011–2012 THMISs.

## Methods

### Study design and population

This was a cross-sectional study, which was conducted using secondary data from the 2003–2004 THIS, as well as the 2007–2008 and 2011–2012 THMISs, which included among other things, HIV serostatus testing. In all three cross-sectional surveys, nationally representative data were collected at regular intervals at 2003/2004, 2007/2008 and 2011/2012.

### Selection of study participants and data sources

**Fig. 1:**
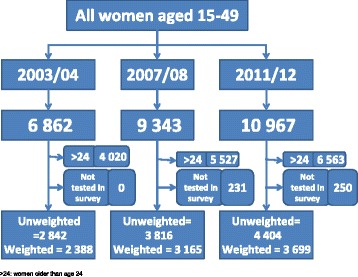
Selection of study participants from the 2003–2004 THIS and the 2007–2008 and 2011–2012 THMISs

Study participants were restricted to young women aged 15–24 years, as this group is considered to be a high-risk population for HIV infection. The final weighted total sample size was 9 252 from the three surveys (see Fig. 1).

### Data collection methods

Prior to each survey and in consultation with stakeholders and donors such as Tanzanian Ministry of Health and ICF International, a Technical Working Group in Tanzania adapted standardised questionnaires from the Demographic and Health Surveys (DHS) Program and the AIDS Indicator Survey to suit the health needs of Tanzania. These questionnaires were then translated into Kiswahili. After obtaining informed consent, the questionnaires were administered to participating individuals. Among other things, the questionnaires captured information on socio-demographic characteristics (age, gender, marital status, education, residence, religion, employment, asset ownership); biological characteristics (having a sexually transmitted infection [STI] in the last 12 months); and other factors such as receiving HIV test results and antenatal care (ANC) for recent births.

### Definition of outcome variables

The outcome variable is the proportion of young women who reported that they were tested for HIV and received test results in the 2 years preceding the surveys.

### Independent variables

The independent variables explored in this study are summarised in Table 1. These include the following socio-demographic variables: respondent’s age (15–19, 20–24 years); place of residence (rural, urban); administrative zone (Central, Lake, Northern, Eastern, Western, South West Highlands, Southern Highlands, Southern); marital status (never married or ever married); employment status (employed, not employed) and number of sexual partners over lifetime (0, 1, 2, >2). Those with a missing value in this last category (*n* = 20) were assumed to have >2 partners.

**Table 1:** Percentage distribution of various characteristics of female respondents aged 15–24 in the 2003–2004 THIS and the 2007–2008 and 2011–2012 THMISs

Characteristics	2003 (*n* = 2 388)	2007 (*n* = 3 165)	2011 (*n* = 3 699)
Age (years)			
15–19	51.7	53.1	55.9
20–24	48.3	46.9	44.1
Residence			
Urban	34.2	26.1	26.6
Rural	65.8	73.9	73.4
Region (zone)			
Central	10.8	8.0	9.0
Lake	25.4	28.9	28.9
Northern	13.0	13.0	11.8
Eastern	24.4	15.5	16.0
Western	8.1	11.6	9.6
South West Highlands	9.3	9.6	10.2
Southern Highlands	7.8	7.5	9.7
Southern	5.2	5.8	4.8
Education level			
No education	17.2	17.9	13.3
Primary	71.3	70.4	59.5
Secondary and above	11.6	11.6	27.1
Marital status			
Never married	51.2	51.2	55.2
Ever married	48.8	48.9	44.8
Employment status			
Not employed	37.0	40.3	36.2
Employed	63.0	59.7	63.8
Number of sexual partners over lifetime			
0	29.8	31.0	31.7
1	33.9	34.0	36.0
2	18.1	20.0	18.1
3+	18.1	14.9	14.2
Blood test result			
HIV-negative	96.0	96.2	97.2
HIV-positive	4.0	3.8	2.8
Had STI in last 12 months			
No	98.6	98.4	98.2
Yes	1.4	1.6	1.8
Giving birth and receiving ANC			
No birth in the 2 years preceding the survey	66.4	68.2	68.1
Gave birth in the 2 years preceding the survey and received ANC	30.6	31.3	30.9
Gave birth in the 2 years preceding the survey but did not receive ANC	3.0	0.6	0.9

Zones rather than administrative regions were used in order to have consistency across the surveys. Between 2003 and 2012, some of the regions were split to form new districts and regions, and thus the 2011–2012 survey had more administrative regions than the 2003–2004 survey. All regions, however (new and old), belong to the same zones and the geographical coverage of the zones has remained consistent throughout the surveys. Composition of the administrative regions in their respective zones is as follows: Eastern (Morogoro, Coastal, Dar es Salaam); Northern (Kilimanjaro, Tanga, Arusha); Lake (Mwanza, Geita, Mara, Simiyu, Shinyanga); Central (Dodoma, Manyara, Singida); Western (Kigoma, Tabora); South West Highlands (Katavi, Rukwa, Mbeya); Southern Highlands (Iringa, Njombe, Ruvuma) and Southern (Lindi, Mtwara).

The biological variables explored included: reported having an STI or symptoms associated with STIs in the last 12 months, and giving birth and receiving ANC in the past 2 years preceding the surveys. Women who had given birth in the 2 years prior to the survey were asked whether they received any ANC (any number of visits) from any provider for their most recent birth. Respondents were grouped accordingly: those who did not give birth in the past 2 years, those who gave birth and received ANC, and those who gave birth but did not receive ANC.

### Statistical analysis

Statistical analyses were performed using Stata version 12.0, StataCorp LP, Texas. To evaluate the trend in the uptake of HIV testing across the three surveys, we performed descriptive analyses of HIV testing stratified by the selected variables. Analyses were done separately for the periods 2003–2007, 2008–2011 and 2003–2011. Pooled logistic regression models were run for each of the two sub-periods (2003–2004 to 2007–2008 and 2007–2008 to 2010–2011) to determine whether the year of the survey was associated with HIV testing uptake independently from the other factors.

Multivariate decomposition models were used to assess effects of various participants characteristics to the changes in uptake of HIV testing. Generally the model portions changes of outcome into two parts, namely changes in characteristics of the variables over time and seasonal changes. In this case, the effect of the changes in uptake of HIV testing will be explained the changes in the characteristics of the participants between the surveys (i.e. endowments, explained variation [E] and seasonal changes (differences in coefficients unexplained variation, C). For example, changes in HIV testing could be due to differences in the increased proportion of urban dwellers participants who are likely to test for HIV or, due to the increased availability of HIV testing kits between surveys.

All analyses performed in this study were weighted for probability sampling and non-response, as is standard in all surveys that are part of the DHS Program. All associations were deemed statistically significant at a cut-off *P*-value of less than 0.05. Complex sampling (multi-stage sampling and stratification) and 95 % confidence intervals were considered.

### Ethical considerations

THIS and THMIS received approval from the Tanzania National Institute of Medical Research, the Institutional Review Board of ICF International and the US Centers for Disease Control and Prevention. All adult respondents gave informed consent. As part of the DHS Fellowship, the authors submitted a proposal to the DHS Program/ICF International and received permission to download and use the data for this study. The DHS Program authorised data access and the data were used solely for the purpose of the current study.

## Results

### Trends in uptake of HIV testing

**Fig. 2:**
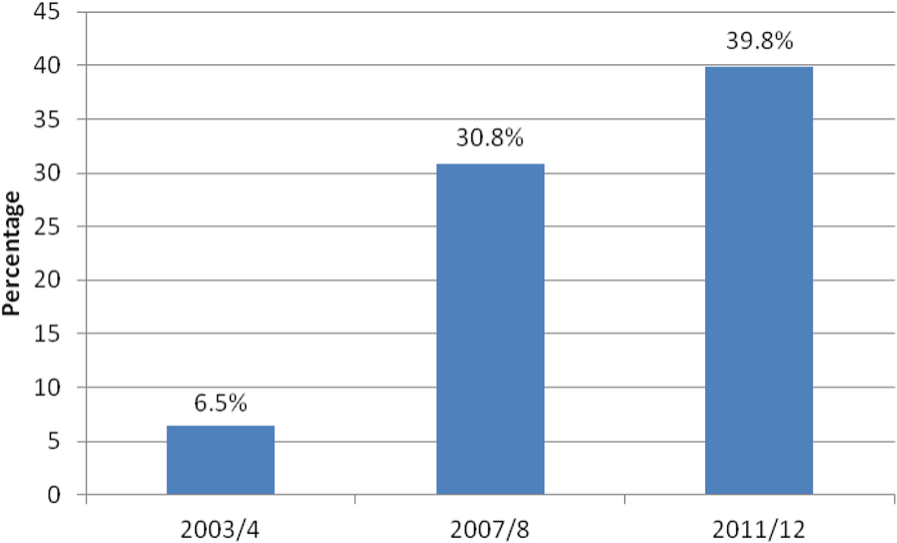
Percentage of women aged 15–24 who tested for HIV and received results in the 2003–2004 THIS and the 2007–2008 and 2011–2012 THMISs

Figure 2 depicts the trends in the study participants’ uptake of HIV testing and receiving results in the 2 years preceding the survey (for the three consecutive surveys). Uptake of HIV testing increased remarkably from 7 % in 2003–2004 to 31 % in 2007–2008 to 40 % in 2011–2012.

### Characteristics of the study participants

Table 1 shows the various characteristics of the study participants. In all three surveys, women aged 15–19 outnumbered those aged 20–24, and at least 65 % of the participants were living in rural areas. The majority of women had a primary level of education (71 % in 2003–2004, 70 % in 2007–2008 and 60 % in 2011–2012). Concerning the lifetime number of sexual partners in 2007, 31 % of women reported no sexual partners, 34 % reported one lifetime sexual partner and 20 % reported two lifetime sexual partners. There was a higher proportion of women reporting three or more lifetime sexual partners (18 %) in the 2003–2004 survey compared with the 2007–2008 and 2011–2012 surveys (both 14.9 %).

Around two-thirds of the women were employed (63 % in 2003–2004, 60 % in 2007–2008 and 64 % in 2011–2012). The percentage of women who were living with HIV declined from 4 % in 2003–2004 to 3 % in 2011–2012. Across the three surveys, around two-thirds of the women had not given birth in the 2 years preceding the survey; the figures were 66, 68 and 68 % in 2003–2004, 2007–2008 and 2011–2012, respectively. Across the three surveys, about one-third of the women (31 %) reported giving birth and receiving ANC in the 2 years preceding the survey.

### Changes in uptake of HIV testing in relation to participants’ characteristics

Table 2 summarises the changes in uptake of HIV testing in relation to the participants’ characteristics. In order to assess the change, the analysis of HIV testing was divided into two phases: between the 2003–2004 and 2007–2008 surveys (phase 1), and between the 2007–2008 and 2011–2012 surveys (phase 2). Overall, the uptake of HIV testing increased by 24 percentage points in phase 1 and 9 percentage points in phase 2, reflecting a more rapid rate of change in the former phase than in the latter. Uptake of HIV testing increased both among women aged 15–19 and 20–24 years. However, the change was greater among women aged 20–24 years. Among the women residing in urban areas, uptake of HIV testing increased by 27 percentage points in phase 1, which is nearly six times than the 5-percentage point-increase in phase 2.

**Table 2:** Trends in uptake of HIV testing among women aged 15–24 (percentage who have been tested for HIV and received results in the 2 years preceding the survey, by characteristics, in the 2003–2004 THIS and the 2007–2008 and 2011–2012 THMISs)

				Percentage point difference in HIV testing		
Characteristics	2003/04 (*n* = 2 388)	2007/08 (*n* = 3 165)	2011/12 (*n* = 3 699)	Phase 1 2007–2003	Phase 2 2011–2007	2011–2003
Age (years)						
15–19	5.8	21.3	27.1	15.5	5.8	21.3
20–24	7.3	41.5	55.9	34.2	14.4	48.6
Residence						
Urban	13.0	39.9	44.6	26.9	4.7	31.6
Rural	3.2	27.5	38.1	24.3	10.6	34.9
Region (zone)						
Central	5.2	25.8	37.9	23.7	6.1	29.8
Lake	5.3	23.9	36.5	26.8	7.3	34.1
Northern	8.3	33.1	37.7	28.4	8.7	37.1
Eastern	11.9	39.1	42.9	21.4	15.6	37.0
Western	5.8	40.5	45.0	25.4	11.2	36.6
South West Highlands	3.9	18.9	32.1	15.0	13.2	28.2
Southern Highlands	3.1	41.7	48.4	38.6	6.7	45.3
Southern	1.6	30.0	47.4	28.4	17.4	45.8
Education level						
No education	2.5	25.2	33.5	22.7	8.3	31.0
Primary	5.9	30.5	40.1	24.6	9.6	34.2
Secondary and above	16.3	40.7	42.5	24.4	1.8	26.2
Marital status						
Never married	6.8	20.2	27.9	13.4	7.7	21.1
Ever married	6.3	41.8	54.5	35.5	12.7	48.2
Employment status						
Not employed	8.7	25.1	34.0	16.4	8.9	25.3
Employed	5.3	34.6	42.9	29.3	8.3	37.6
Number of sexual partners over lifetime						
0	4.5	9.6	13.7	5.1	4.1	9.2
1	7.9	38.5	48.3	30.6	9.8	40.4
2	7.5	40.7	57.4	33.2	16.7	49.9
3+	6.4	43.9	54.4	37.5	10.5	48.0
Blood test result						
HIV-negative	6.6	30.5	39.3	23.9	8.8	32.7
HIV-positive	6.5	36.3	57.0	29.8	20.7	50.5
Had STI in last 12 months						
No	6.4	30.5	39.4	24.1	8.9	33.0
Yes	17.2	49.2	62.2	32.0	13.0	45.0
Giving birth and receiving ANC						
No birth in the 2 years preceding the survey	8.3	22.6	26.5	14.3	3.9	18.2
Gave birth and received ANC in the 2 years preceding the survey	2.8	48.6	69.0	45.8	20.4	66.2
Gave birth but didn't receive ANC in the 2 years preceding the survey	6.9	25.3	44.9	18.4	19.6	38.0
Total	6.6	30.8	39.8	24.2	9.0	33.2

In the surveys, the change in uptake of HIV testing was greater among married women compared with unmarried women, and also greater among HIV-positive women compared with HIV-negative women. Compared with women who did not give birth in the 2 years preceding the survey, the change in HIV testing was greater among those who gave birth and received ANC (46 percentage points versus 14 percentage points in phase 1 and 20 percentage points versus 4 percentage points in phase 2). The change in uptake of HIV testing in phase 1 was much greater among women with three or more lifetime sexual partners, at 38 percentage points, than among women with no sexual partners, at 5 percentage points. In phase 2, this difference was smaller.

### Factors associated with uptake of HIV testing across the surveys

Table 3 shows the results from the pooled logistic regression models for factors associated with uptake of HIV testing across the two survey phases. Of the factors examined, those found to be associated with a higher likelihood of getting testing for HIV and receiving results included having an education (primary or secondary), ever being married, having had at least one lifetime sexual partner and receiving ANC. Having had an STI was also associated with uptake of HIV testing, but the association was not statistically significant.

**Table 3:** Pooled multivariate logistic regression of factors associated with uptake of HIV testing among women aged 15–24 in the 2003–2004 THIS and the 2007–2008 and 2011–2012 THMISs

Surveys	Phase 1	Phase 2
	2003/04–2007/08	2007/08–2011/12
	HIV testing uptake	
	*OR* (95 % *CI*)	*OR* (95 % *CI*)
Characteristics		
Survey year	8.71 (6.80–11.15)	1.34 (1.11–1.61)
Age (years)		
15–19	1.0	1.0
20–24	1.02 (0.80–1.30)	1.27 (1.07–1.50)**
Residence		
Urban	1.0	1.0
Rural	0.46 (0.36–0.58)***	0.53 (0.44–0.63)***
Region (zone)		
South West Highlands	1.0	1.0
Central	1.35 (0.83–2.19)	1.50 (1.06–2.14)**
Lake	1.34 (0.89–2.02)	1.15 (0.87–1.53)
Northern	2.13 (1.42–3.19)***	1.94 (1.34–2.83)**
Eastern	1.89 (1.25–2.85)**	1.74 (1.27–2.39)**
Western	3.15 (2.06–4.83)***	2.65 (1.85–3.80)***
Southern Highlands	2.08 (1.34–3.23)**	2.19 (1.59–3.02)***
Southern	1.44 (0.84–2.46)	1.72 (1.07–2.77)**
Education level		
No education	1.0	1.0
Primary	1.90 (1.46–2.46)***	1.99 (1.57–2.52)***
Secondary and above	5.46 (3.76–7.94)***	4.81 (3.48–6.63)***
Marital status		
Never married	1.0	1.0
Ever married	1.49 (1.15–1.92)**	1.36 (1.13–1.64)***
Employment status		
Not employed	1.0	1.0
Employed	0.99 (0.79–1.26)	1.09 (0.91–1.32)
Number of sexual partners over lifetime		
0	1.0	1.0
1	4.44 (3.21–6.15)***	3.88 (3.09–4.88)***
2	4.74 (3.30–6.82)***	4.59 (3.52–5.99)***
3+	4.62 (3.12–6.84)***	4.77 (3.55–6.40)***
Blood test result		
HIV-negative	1.0	1.0
HIV-positive	0.86 (0.55–1.35)	1.14 (0.74–1.75)
Had STI in last 12 months		
No	1.0	1.0
Yes	1.62 (0.97–2.77)	1.54 (0.97–2.44)
Giving birth and receiving ANC		
No birth in the 2 years preceding the survey	1.0	1.0
Gave birth and received ANC in the 2 years preceding the survey	1.47 (1.18–1.83)**	3.10 (2.60–3.69)***
Gave birth but did not receive ANC in the 2 years preceding the survey	1.22 (0.48–3.10)	1.51 (0.83–2.75)

* Significant at *P* < 0.05; ** Significant at *P* < 0.01; *** Significant at *P* < 0.001

The results from the multivariate decomposition regression models are shown in Tables 4 and 5. According to the models, in 2003–2004 compared with 2007–2008, changes in the study participants’ characteristics would have resulted in a 5.2 % decline in overall HIV testing in the absence of any changes in the coefficients (seasonal changes).

**Table 4:** Decomposition changes in HIV testing among women aged 15–24 in the 2003–2004 THIS and the 2007–2008 and 2011–2012 THMISs

HIV testing		Due to differences in characteristics (E)		Due to differences in coefficients (C)	
		Coefficient	Percent	Coefficient	Percent
Age (years)	15–19	1.0		1.0	
	20–24	−0.0001	−0.0288	0.0067	2.7657
Residence	Urban	1.0		1.0	
	Rural	−0.0089***	−3.6661	0.0461**	19.0522
Region (zone)	South West Highlands	1.0		1.0	
	Central	−0.0024*	−0.9758	0.0002	0.8422
	Lake	0.0013	0.5495	−0.0014	−0.5948
	Northern	−0.0057***	−0.0035	0.0015	0.6041
	Eastern	−0.0064**	−2.6346	0.0033	2.1948
	Western	0.0079***	3.2746	0.0049	2.009
	Southern Highlands	−0.0005***	−0.2239	0.0084	3.4742
	Southern	−0.0005	0.2094	0.0092	3.8014
Education level	No education	1.0		1.0	
	Primary	−0.001***	−0.4253	−0.0137	−5.6564
	Secondary and above	0.0003***	0.1225	0.0003	−0.1093
Marital status	Never married	1.0		1.0	
	Ever married	0.0001**	0.0197	−0.0042	−1.7221
Employment status					
	Not employed	1.0		1.0	
	Employed	−0.0006	−0.2258	0.0092	3.8152
Number of sexual partners over lifetime	0	1.0		1.0	
	1	0.0001***	0.0481	0.0156	6.4606
	2	0.0052 ***	2.1621	0.0121	5.0033
	3+	−0.0091 ***	−3.7754	0.0170	7.0277
Blood test result	HIV-negative	1.0		1.0	
	HIV-positive	0.0004	0.0145	0.0012	0.4751
Had STI in last 12 months					
	No	1.0		1.0	
	Yes	0.0001	0.0205	−0.0022	−0.8932
Giving birth and receiving ANC <2 years preceding the survey					
	No birth	1.0		1.0	
	Birth and ANC	0.0009***	0.3740	0.0793***	32.7662
	Birth but no ANC	−0.0002	−0.0955	0.0001	0.0449
Constant				0.0597***	24.6542
Total			−5.2563**		105.2624***

* Significant at *P* < 0.05; ** Significant at *P* < 0.01; *** Significant at *P* < 0.001

**Table 5:** Decomposition changes in HIV testing among women aged 15–24 in the 2007–2008 and 2011–2012 THMISs

HIV testing		Due to differences in characteristics (E)		Due to differences in coefficients (C)	
		Coefficient	Percent	Coefficient	Percent
Age (years)	15–19	1.0		1.0	
	20–24	−0.0019***	−2.0725	0.0320**	35.2923
Residence	Urban	1.0		1.0	
	Rural	0.0004**	0.4002	0.0330	36.3824
Region (zone)	South West Highlands	1.0		1.0	
	Central	0.0005	0.5898	−0.0030	−3.3221
	Lake	−0.0001	−0.0116	−0.0072	−7.9518
	Northern	−0.0011**	−1.2432	−0.0074	−8.1773
	Eastern	0.0003	0.3251	−0.0119	−13.0552
	Western	−0.0032**	−2.5389	−0.0144**	−15.9034
	Southern Highlands	0.0024**	2.6182	−0.0053	−5.5294
	Southern	−0.0012**	−1.3627	0.0029	3.2135
Education level	No education	1.0		1.0	
	Primary	−0.0143***	−15.8031	0.0124	13.6283
	Secondary and above	0.0423***	46.6533	−0.0022	−2.4257
Marital status	Never married	1.0		1.0	
	Ever married	−0.0022**	−2.4582	−0.0032	−3.5296
Employment status					
	Not employed	1.0		1.0	
	Employed	0.0001	0.0118	−0.0109	−12.1
Number of sexual partners over lifetime		1.0		1.0	
	0	1.0		1.0	
	1	0.0039***	4.3212	−0.0248	−27.3214
	2	−0.0043***	−4.7523	−0.0123	−13.5252
	3+	−0.0015***	−1.6056	−0.0127	−14.0341
Blood test result					
	HIV-negative	1.0		1.0	
	HIV-positive	−0.0009	−0.9805	0.0043	4.7497
Had STI in last 12 months					
	No	1.0		1.0	
	Yes	0.0003	0.3193	0.0008	0.8537
Giving birth and receiving ANC attendance <2 years preceding the survey					
	No birth	1.0		1.0	
	Birth and ANC	−0.0007***	−0.7741	0.0395***	43.5231
	Birth but no ANC	0.0004**	0.4654	0.0007	0.7629
Constant				0.6022	66.3713
Total			22.1***		77.9***

* Significant at *P* < 0.05; ** Significant at *P* < 0.01; *** Significant at *P* < 0.001

Comparing the 2007–2008 and 2011–2012 surveys, the changes in endowments contributed to 22 % of the changes in the uptake of HIV testing, whereas 78 % of the changes in the uptake of HIV testing was attributed to coefficients. Both endowments and coefficients were statistically significant in both multivariate decomposition models. Having any level of education versus having none made it much more likely that women would get tested for HIV. The increase in the proportion of women with some education should have made it more likely for women to be tested, regardless of any changes in effects of other characteristics.

## Discussion

### Trends in uptake of HIV testing

The present study demonstrated a rapid increase in the uptake of HIV testing among women aged 15–24 in Tanzania. Nearly 40 % of the women aged 15–24 who were included in the 2011–12 survey had been tested for HIV and received their test results, which was six times higher than the percentage of women who were tested for HIV in the 2003–2004 survey. A greater increase in the uptake of HIV testing occurred between the 2003–2004 and 2007–2008 surveys as compared to between the 2007–2008 and 2011–2012 surveys. This increase could be attributable to the wider availability of rapid HIV testing kits (which lowers the cost of testing), the rolling out of free ART which started in December 2004, as well as national campaigns to promote HIV testing [7, 25]. On the other hand, the increase in the uptake of HIV testing may also be due to the enactment of the 2008 law forbidding discrimination against people living with HIV (PLWHA), as well as the increased availability and access to HIV testing services such as provider-initiated testing and counselling, and community/family counselling and testing [7, 26]. The increase in HIV testing translates into an increased proportion of people who are aware of their HIV status and thus possibly changing their behaviours in ways that can reduce the risk of HIV transmission. Therefore, higher levels of HIV testing uptake are needed in a test-and-treat model if a reduction in HIV incidence is to be realised [27–29].

### Determinants of HIV testing uptake

This study found numerous factors that are associated with uptake of HIV testing. The odds of HIV testing uptake were higher among women with primary and/or secondary education compared with women without any formal education. This finding is in agreement with a previous study conducted among Ghanaian women, which showed that a higher level of education was strongly correlated with uptake of HIV testing [17]. A possible explanation for this could be that higher educational attainment provides more opportunities to clearly understand HIV infection and prevention. Moreover, women with more education are more likely to be employed and have higher earnings as compared to their less educated counterparts [30, 31], and therefore have better access to HTC services. Until about 2004, most HTC centres would charge clients a fee for services.

Marital status was shown to be associated with uptake of HIV testing in this study. Married women were more likely to undergo HIV testing than unmarried women. Similar findings have been documented in other studies [17, 32]. In our study, the greater uptake of HIV testing among married women could be due to faith-based institutions advocating for the importance of having HIV testing before marriage in Tanzania.

Compared with women without sexual partners, the odds of HIV testing uptake were higher in those women with at least one lifetime sexual partner, a finding that is consistent with previous studies conducted in Tanzania, as HIV infection is predominantly transmitted through sexual contact in the country [25].

The present study also found that receiving ANC was an important determinant for HIV testing. Women who had given birth in the 2 years preceding the surveys and received ANC had increased odds of getting HIV tested compared with women who did not give birth. No association of HIV testing uptake was noted among women who had given birth but did not receive ANC. This finding is in agreement with previous reports in Tanzania [33]. To achieve PMTCT, women are required to get tested in order to receive ART and also to make decisions about breastfeeding and family planning practices. This requirement could explain the higher uptake of HIV testing among women who received ANC.

Analysis showed a difference in HIV testing uptake in relation to STI status. Having a STI or symptoms of an STI was associated with increased odds of HIV testing. Although the association was not statistically significant, it may be clinically meaningful. An association between having a STI and HIV testing uptake has also been reported elsewhere [34]. STIs increase the risk of HIV transmission and are often transmitted along with HIV. Individuals attending STI clinics are thus more likely to be counselled and eventually tested for HIV, which could explain the higher uptake of HIV testing among this group.

This study also revealed that uptake of HIV testing changes with age. Women aged 20–24 years had increased odds of getting tested for HIV as compared with women aged 15–19 years. This finding is consistent with previous studies [23, 25, 35]. In addition, the study found that women who live in urban areas were more likely to get tested for HIV compared with rural women. Similar findings have also been reported elsewhere [36, 37]. Urban areas offer greater access to HCT services and thereby increased communication about HIV compared with rural areas. We also found zonal variations in the uptake of HIV testing across the surveys. This could be explained by the regional variations in the availability of HCT services across the zones.

### Decomposition of the changes in uptake of HIV testing

Decomposition analyses distinguished the sources in the changes in HIV testing uptake. Changes in the characteristics of women who had different levels of education, number of ANC visits and places of residence contributed to an observable change in the uptake of HIV testing. These factors have also been associated with an increased uptake of HIV testing in previous studies [17, 33, 37]. For example, during phase 1, a decrease in the proportion of women with primary education or an increase in the proportion of women residing in rural areas would have resulted in a decrease in the uptake of HIV testing in the absence of the coefficient. Although changes in of the study participant’s characteristics contributed to an increased uptake of HIV testing (22 %), most of the HIV testing uptake was due to changes in the coefficient (78 %). For example, receiving ANC significantly contributed to changes in the uptake of HIV testing in both the first and second phases. On the other hand, the change in the uptake of HIV testing may be attributed to the availability of different testing options such as couple testing, mobile testing and school-based and workplace testing [7].

### Strengths and limitations of the study

Unlike standard logistic regression-based approaches that rely on individual-level data, multivariate regression decomposition in HIV testing provides an opportunity for detailed explanations for the differences in the changes in HIV testing across the surveys. Because of the limited information in DHS survey data; we were not able to assess the true effect of the HIV interventions performed across the survey periods as this data was not available in the dataset. For this reason, the presence of more data might have also influenced the observed changes in HIV testing. The use of self-reported data may have introduced social desirability bias and thereby affected the reported findings.

## Conclusions

In conclusion, uptake of HIV testing increased remarkably during the time period of the three surveys. Although there is a remarkable increase in the uptake of HIV testing, the increment among participant’s characteristics is to a large extent explained by the study participants receiving ANC. This is a reflection of the rapid expansion of HIV testing services into ANC in order to increase PMTCT. Knowing one’s HIV status is the gateway to HIV treatment and prevention. However, the expansion in HIV testing has been greater among women with high-risk characteristics, and thus has become more targeted. Until testing is universal, an effective expansion strategy would be to prioritize those groups most likely to be infected.

## Additional file

Additional file 1: Multilingual abstract in the six official working languages of the United Nations. (PDF 662 kb)

## Abbreviations

AIDS: Acquired immune deficiency syndrome
ANC: Antenatal care
ART: Antiretroviral therapy
DHS: Demographic and health surveys
HIV: Human immunodeficiency virus
HTC: HIV testing and counselling
PMTCT: Prevention of mother-to-child transmission
SSA: Sub-Saharan Africa
STI: Sexually transmitted infection
THIS: Tanzania HIV/AIDS indicator survey
THMIS: Tanzania HIV/AIDS and malaria indicator survey
