# Supplementary material for: Factors associated with changes in uptake of HIV testing among young women (aged 15–24) in Tanzania from 2003 to 2012
Source: Infect Dis Poverty. 2016 Sep 6;5(1):92. doi: 10.1186/s40249-016-0180-3 (PMC5011841; doi:10.1186/s40249-016-0180-3)
Supplement: Additional file 1: — Multilingual abstract in the six official working languages of the United Nations. (PDF 662 kb) [file 40249_2016_180_MOESM1_ESM.pdf]

Translation of the abstract into the six official working languages of the United Nations

العوامل المرتبطة بالتغيرات في الإقبال على اختبار فيروس نقص المناعة المكتسبة عند النساء التي تتراوح أعمارهم بين 15-24 في تنزانيا مابين العام 2003 و 2012

مايكل مهاندي, رون فيميمون, حبيب رامادهاني.
Michael J. Mahande, Rune N. Phimemon, Habib O. Ramadhani

الملخص
خلفية الموضوع: بحثت هذه الدراسة في العوامل المرتبطة بالتغيرات في الاقبال على اختبار فيروس نقص المناعة المكتسبة بين الشابات في تنزانيا استنادا على تحليل بيانات استبيان تانزانيا بين عامي 2003- 2004 و استبيان نقص المناعة المكتسبة والملاريا للأعوام 2007-2008 و2011-2012.
المنهجية: تتكون العينة التي تمت دراستها من شابات تتراوح أعمارهن بين 15-24 سنة في وقت إجراء الاستبيان. وقد تم استخدام تحليل متعدد المتغيرات لتقييم العوامل المرتبطة بالتغيرات في الاقبال على اختبار فيروس نقص المناعة المكتسبة بين الأعوام 2003-2004 و2007-2008، وبين استبيانات الأعوام 2007-2008 و2011-2012.
النتائج: تبين أن الاقبال على اختبار فيروس نقص المناعة المكتسبة عند العينة التي تمت دراستها كان 7٪ بين عامي 2003-2004 و 31٪ في 2007-2008 و 40٪ في 2011-2012. وكان للفترة الزمنية للدراسة تأثير كبير على الإقبال على اختبار فيروس نقص المناعة المكتسبة بمعزل عن المتغيرات الأخرى. وقد تبين ان هناك ارتباط كبير بين الخصائص التالية والاقبال الأكبر على اختبار فيروس نقص المناعة المكتسبة في الاستبيان للعينة التي تتراوح أعمارهم بين 20-24: مستوى التعليم (الابتدائي والثانوي) و ان سبق لهم الزواج في أي وقت مضى أم لا و ان كان لديهم على الأقل شريك واحد و وجود عدوى تنتقل بالاتصال الجنسي أو أحد الأعراض المصاحبة وتلقي الرعاية السابقة للولادة.
الخاتمة: ارتبطت التغيرات في خصائص المشاركين في الدراسة في استبيان عام 2003-2004 مقارنة مع الاستبيان عامي 2007-2008 بانخفاض في الاقبال على اختبار فيروس نقص المناعة المكتسبة. وبمقارنة استبيان عامي 2007-2008 مع استبيان عامي 2011-2012 يتبين ان التغيرات في خصائص المشاركين قد ساهمت ب 22٪ من التغيرات في الاقبال على اختبار فيروس نقص المناعة المكتسبة في حين ان 78٪ من التغييرات تعود الى عوامل أخرى ذات صلة.

Translated from English version into Arabic by Randa82, through

TRANSLATORS
WITHOUT BORDERS

2003-2012 年坦赞利亚年轻女性（15-24 岁）接受 HIV 检测的变化及影响因素

Michael J. Mahande, Rune N. Phimemon, Habib O. Ramadhani

摘要
引言：本研究基于坦赞利亚 2003-2004 年的 HIV/AIDS 指标调查、2007-2008 年及 2011-2012 年的 HIV/AIDS 和疟疾指标调查的数据，探索坦赞利亚年轻女性接受 HIV 检测的变化情况及影响因素。
方法：本研究人群为调查时为 15-24 岁的年轻女性。采用多因素分解法比较 2003-2004 年和 2007-2008 年、2007-2008 年和 2011-2012 年接受 HIV 检测变化的影响因素。
结果：2003-2004 年、2007-2008 年和 2011-2012 年研究人群接受 HIV 检测的比例分别为 7%、31%和 40%。调查时段对 HIV 检测的接受性有很大影响，且独立于其他因素。HIV 检测接受增加的显著性因素包括年龄（20-24 岁）、教育水平（小学和中学）、已婚、至少有一个终生性伴侣、曾有性病感染史或相关症状及产前保健。
结论：与 2007-2008 年相比，2003-2004 年人群的特征差异与 HIV 检测接受的较低有关。与 2007-2008 年相比，2011-2012 年人群的特征差异对 HIV 检测接受的变化的贡献占到 22%，其余 78%归因于共效应。

Translated from English version into Chinese by Qian Men-Bao

CDC CHINESE CENTER FOR DISEASE CONTROL AND PREVENTION
NATIONAL INSTITUTE OF PARASITIC DISEASES

Les facteurs liés aux changements d'assimilation du dépistage du VIH chez les jeunes femmes (âgées 15–24) en Tanzanie de 2003 à 2012

Michael J. Mahande, Rune N. Phimemon, Habib O. Ramadhani

Résumé
Historique: Cette étude a étudié les facteurs liés aux changements d'assimilation du dépistage du VIH chez les jeunes femmes en Tanzanie, se basant sur une analyse des données d'une enquête sur les indicateurs de VIH/SIDA 2003-2004 en Tanzanie, et du VIH/SIDA 2007-2008 et 2001-2012 en Tanzanie et des enquêtes sur les indicateurs du paludisme.
Méthodes: La population étudiée était composée de jeunes femmes âgées de 15-24 ans au moment de l'enquête. L'analyse multivariée de décomposition a été utilisée pour évaluer les facteurs liés aux changements d'assimilation du dépistage du VIH entre les enquêtes de 2003-2004 et 2007-2008, et entre les enquêtes de 2007-2008 et 2011-2012.
Résultats: L'assimilation du dépistage du VIH de la population étudiée était de 7% en 2003-2004, de 31% en 2007-2008 et de 40% en 2011-2012. La période de l'enquête a eu un effet important sur l'assimilation du dépistage du VIH indépendant des autres co-variables. Les caractéristiques qui ont été significativement liées à un risqué plus élevé de l'assimilation du dépistage VIH dans les enquêtes

étaient l'âge (20-24), le niveau d'éducation (primaire et secondaire), marié, avoir eu au moins un partenaire dans leur vie sexuelle, ayant eu une infection sexuellement transmissible ou des symptômes associés, et recevant des soins prénatals.
Conclusions: Les changements dans les caractéristiques des participants de l'étude dans l'enquête 2003-2004 par rapport à l'enquête 2007-2008 étaient liés à la diminution de l'assimilation du dépistage VIH. En comparant l'enquête 2007-2008 à l'enquête 2011-2012, on constate que les changements des caractéristiques des participants ont contribué à 22% des changements d'assimilation du dépistage VIH, alors que 78% des changements ont été attribués aux coefficients.

Translated from English version into French by ishaklamia, through

Факторы, связанные с изменениями в проведении тестирования на ВИЧ среди молодых женщин (в возрасте от 15 до 24 лет) в Танзании за период с 2003 по 2012 гг.

Майкл Дж. Маханде, Руне Н. Фимемон, Хабиб О. Рамадхани/Michael J. Mahande, Rune N. Phimemon, Habib O. Ramadhani

Аннотация
История вопроса: В данном исследовании изучали факторы, связанные с изменениями в проведении тестирования на ВИЧ среди молодых женщин в Танзании. Исследование проводили на основе анализа данных Обзора индикаторов ВИЧ/СПИД в Танзании за период с 2003 по 2004 гг., и Обзоров индикаторов ВИЧ/СПИД и малярии в Танзании за период с 2007 по 2008 гг. и с 2011 по 2012 гг.
Методы: Группа исследования включала молодых женщин, которые на момент исследования достигли возраста от 15 до 24 лет. Для оценки факторов, связанных с изменениями в проведении тестирования на ВИЧ, использовали многовариантный декомпозиционный анализ данных обзоров за период с 2003 по 2004 и с 2007 по 2008 гг, и за период с 2007 по 2008 и с 2011 по 2012 гг.
Результаты: Проведение тестирования на ВИЧ в группе исследования составило 7% в 2003–2004, 31% в 2007–2008 и 40% в 2011–2012. Временной период обзора оказывал значительное действие на проведение тестирования на ВИЧ независимо от других ковариантов. Характеристиками, имеющие значительное влияние на повышение частоты проведения тестирования на ВИЧ в рамках изучения обзоров, были возраст (20-24), уровень образования (начальное образование и среднее образование), семейное положение, наличие по меньшей мере одного полового партнера, наличие инфекции, передающейся половым путем, или связанных с ними симптомов, и прохождение дородового наблюдения.
Выводы: Изменения характеристик участниц исследования в обзорах 2003-2004 гг, которые сравнивали с аналогичными изменениями в 2007-2008 гг., были связаны со снижением частоты проведения тестирования на ВИЧ. Сравнение обзора за 2007–2008 гг с обзором за 2011–2012 гг. показало, что изменения характеристик участниц исследования составили 22% от изменений в частоте проведения тестирования на ВИЧ, тогда как 78% изменений относились к коэффициентам.

Translated from English version into Russian by Helga_Sh, through

Factores asociados a la realización de pruebas de VIH en mujeres jóvenes (entre 15 y 24 años) del 2003 al 2012 en Tanzania

Michael J. Mahande, Rune N. Phimemon, Habib O. Ramadhani

Resumen
Introducción: Este estudio examinó los factores asociados con los cambios en la realización de pruebas de VIH en mujeres jóvenes en Tanzania, basándose en un análisis de datos de los Estudios de Indicadores de VIH/SIDA 2003-2004 de Tanzania y de los Estudios de Indicadores de VIH/SIDA y Malaria 2007-2008 y 2011-2012 de Tanzania.
Métodos: La población de estudio estuvo constituida por mujeres jóvenes que al momento del estudio tenían entre 15 y 24 años. Se utilizó un análisis de descomposición multivariante para evaluar los factores asociados con los cambios en la realización de pruebas de VIH entre los estudios de 2003-2004 y de 2007-2008, y entre los estudios de 2007-2008 y de 2011-2012.
Resultados: La realización de pruebas de VIH entre la población del estudio fue de un 7% en 2003-2004, un 31% en 2007-2008 y un 40% en 2011-2012. El periodo de tiempo del estudio tuvo un efecto significativo en la realización de pruebas de VIH independientemente de otras covariables. Las características más asociadas con mayores probabilidades de realización de pruebas de VIH en los estudios fueron: edad (20-24), nivel de educación (primaria y secundaria), estar casada, haber tenido al menos una pareja sexual a lo largo de la vida, haber tenido una enfermedad de transmisión sexual o síntomas relacionados y haber recibido cuidados prenatales.

Conclusiones: Los cambios en las características de los participantes del estudio 2003-2004 en comparación con el estudio 2007-2008 tuvieron relación con una disminución en la realización de pruebas de VIH. Al comparar el estudio 2007-2008 con el estudio 2011-2012, se ve demostrado que los cambios en las características de los participantes contribuyeron con el 22% de los cambios en la realización de pruebas de VIH, mientras que el 78% de los cambios se atribuyeron a coeficientes.

Translated from English version into Spanish by Mpgorgone, through

TRANSLATORS
WITHOUT BORDERS
